# Wrinkle motifs in thin films

**DOI:** 10.1038/srep08938

**Published:** 2015-03-11

**Authors:** Zoe Budrikis, Alessandro L. Sellerio, Zsolt Bertalan, Stefano Zapperi

**Affiliations:** 1 ISI Foundation, Via Alassio 11/c, 10126 Torino, Italy; 2 CNR-IENI, Via R. Cozzi 53, 20125 Milano, Italy; 3 Department of Physics, University of Milano, Via Celoria 16, 20133 Milano, Italy

## Abstract

On length scales from nanometres to metres, partial adhesion of thin films with substrates generates a fascinating variety of patterns, such as ‘telephone cord’ buckles, wrinkles, and labyrinth domains. Although these patterns are part of everyday experience and are important in industry, they are not completely understood. Here, we report simulation studies of a previously-overlooked phenomenon in which pairs of wrinkles form avoiding pairs, focusing on the case of graphene over patterned substrates. By nucleating and growing wrinkles in a controlled way, we characterize how their morphology is determined by stress fields in the sheet and friction with the substrate. Our simulations uncover the generic behaviour of avoiding wrinkle pairs that should be valid at all scales.

Delamination of thin sheets from substrates can be undesirable, for example when plastic coatings peel from glass, but controlled delamination is of interest for applications ranging from flexible electronics^1^ to micro- and nanofluidic devices^2^,^3^. Full realization of these applications requires a deep understanding of the role played by sheet properties and substrate geometry for delamination. In addition, delamination blisters and wrinkles form a rich variety of complex patterns, which are the subject of ongoing research. Examples include circular blisters, “telephone cord” buckles, parallel and radially organized wrinkles, and labyrinth patterns^4^,^5^,^6^,^7^,^8^,^9^,^10^,^11^,^12^,^13^,^14^. Substrate patterning further increases the variety of patterns possible. For example, graphene sheets on substrates with pillars or particles has been experimentally demonstrated to form networks of wrinkles^15^,^16^,^17^,^18^. Previous theoretical work on thin sheet delamination has focused on the properties of a single blister or wrinkle, looking for example at how blister growth is affected by substrate interactions and elastic instabilities^4^,^10^,^12^,^19^. On the other hand, interactions between delaminated regions are fundamental as strikingly illustrated by recent experiments by Yamamoto *et al.*^17^ on graphene sheets.

In those experiments, graphene wrinkles are seen to form pairs that approach each other but do not coalesce^17^ (see Fig. 1), as also confirmed by atomistic simulations^18^. Such avoiding wrinkle pairs are not just a curiosity of graphene sheets on patterned substrates, and are found not only in other thin films and possibly even in macroscopic sheets such as geomembranes used to line landfills, an example of which is shown in Fig. 1. Other examples from the physics and engineering literature are listed in Table 1. Furthermore, similar avoiding patterns are widely observed in “en passant” cracks^20^,^21^, which have a universal shape, independent of material properties^21^. Despite their ubiquity, the physics of avoiding wrinkle pairs is largely unexplored and a quantitative understanding is lacking of when avoiding pairs form and the morphology they take.

Here, we report a simulation study of avoiding and coalescing delamination wrinkles in graphene. The phenomena we describe are not graphene-specific, but we make a case study of graphene because it is an exemplary thin sheet, with much greater stiffness against tension than bending, as well as being fascinating in its own right^22^,^23^,^24^. Furthermore, strain engineering of graphene promises control of electronic, optical and thermal properties^25^,^26^,^27^, so understanding and controlling the morphology of graphene sheets on substrates is essential for building novel devices.

We simulate controlled growth of wrinkles in mono- and multilayer graphene sheets. These simulations reveal the role played by interactions in determining whether wrinkles merge or form avoiding pairs. We quantify how the initial geometry of a pair of growing wrinkles determines its final shape and examine how substrate friction suppresses wrinkle formation. By varying sheet thickness, we demonstrate how the phenomenon of avoiding pair formation is length scale-independent. Our results open a new perspective on how thin films delaminate and point to approaches for controlling film morphology.

## Results

### Coarsegrained model quantitatively reproduces experimental observations of wrinkles on patterned substrates

We are primarily interested in universal phenomena, rather than graphene-specific behavior. We therefore use a linearized model that captures the essential physics of a sheet interacting with a substrate, in which the sheet is a triangular lattice of springs that provide a stretching cost, with pairs of plaquettes joined by hinges that have a harmonic energy favouring a flat sheet, which provides a bending cost. Both stretching and bending energies must be accounted for in order to model accurate wrinkle profiles^17^, in particular, without bending cost, sharp tent-like structures form rather than smooth wrinkles. Similar models have previously proved successful for studying thin sheets^8^,^28^. The sheet is pushed towards to the substrate except at short distances where it is repelled. To reduce spurious edge effects and allow for effects of friction between the sheet and substrate, particles near the sheet edge are coupled to their original lateral positions with harmonic springs, to constrain their lateral (but not vertical) motion.

**Figure 1:**
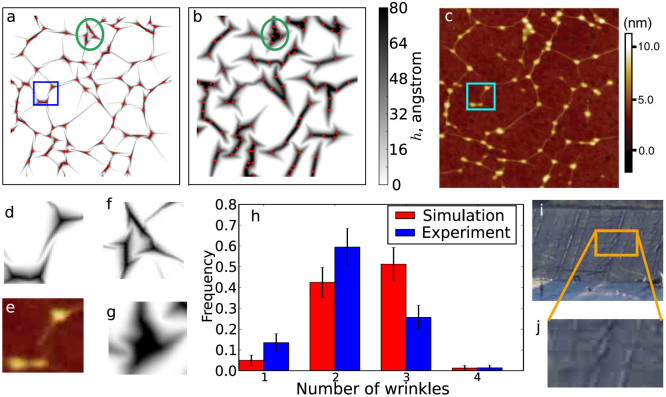
Avoiding wrinkles and substrate patterning-driven delamination occur in both micro- and macroscopic systems, and are reproduced by a coarsegrained model. Simulated 1 × 1 *μ*m^2^ (a) monolayer and (b) 5-layer graphene sheet on a substrate decorated with spherical particles of diameter 8 nm, placed in positions determined by digitization of the experimental micrograph shown in panel (c). Red dots indicate the particle centres. (c) Atomic force micrograph from Ref. 17 (doi: 10.1103/PhysRevX.2.041018), showing a 1 × 1 *μ*m^2^ region of graphene exfoliated onto a substrate decorated with nanoparticles of mean diameter 7.4 nm. (d,e) Close-ups of an example avoiding pair of wrinkles found in experiment and simulation, as highlighted by the blue boxes in panels (a) and (c). (f,g) Close-ups of a region in which the monolayer sheet conforms to the substrate but the 5-layer sheet delaminates, as indicated by the green ellipses in panels (a) and (b). (h) Quantitative agreement between simulation and experiment is evidenced by histograms of the number of wrinkles emanating from each particle, as counted from the images in panels (a) and (b). Error bars correspond to the square root of each count. (i,j) Avoiding wrinkle-like features in geomembranes. The human figure in panel (i) illustrates the scale; panel (j) shows a close-up of the highlighted region, which contains an avoiding pair. Image courtesy Wisconsin Department of Natural Resources^38^.

We verify our model through comparison with experimental studies of graphene sheets deposited on substrates decorated with nanoparticles of diameter ~10 nm, as published recently^17^. We simulate 1 × 1 *μ*m^2^ regions of monolayer and 5-layer graphene, with particles of radius 8 nm placed at positions determined by digitizing the atomic force micrograph of a system with nanoparticle density of 90 *μ*m^−2^^17^. As seen in Fig. 1, the agreement between monolayer simulation and experiment is good, with both systems displaying similar networks of long narrow wrinkles connecting particles. We note that agreement is only expected in the centre of the region simulated, because the experimental image is a sample of a larger region and wrinkles near the edges are affected by particles not shown in the limited field of view.

Importantly, our linearized simulations exhibit avoiding pairs of wrinkles, an example of which is highlighted in Fig. 1(d,e). The pairs are stable and their appearance is robust against changes of simulation details such as substrate interactions. In addition, like Yamamoto *et al.*, we find increasing the thickness of the graphene sheet hinders its conformation to the substrate (compare our Fig. 1 with Fig. 6 of Ref. 17). This is especially apparent in regions where close-spaced particles are positioned on the vertices of a polygon, as in the region indicated in Fig. 1(a,b). As highlighted in Fig. 1(f,g), the sheet in this region “pops up” when its thickness (and hence ratio of bending to stretching costs) increases. In the rest of this paper, we study these two characteristic behaviours in greater detail.

Beyond visual comparisons, we compare simulation and experiment statistically, by measuring the distribution of the number of wrinkles emanating from each particle for the graphene monolayers, as plotted in Fig. 1(h). The agreement is good, although the simulations have a slightly larger mean number of wrinkles per particle (2.48 for simulation, *vs* 2.15 for experiment). We attribute this to lower noise levels in simulation data allowing more wrinkles to be detected.

Features reminiscent of avoiding pairs of wrinkles can be observed also on macroscopic lengthscales, as demonstrated in Fig. 1(i,j), which shows a geomembrane used to line a landfill. That these features are not graphene-specific further supports our choice of coarse grained, rather than atomistic, model.

### Coalescence of growing wrinkles depends on their initial separation

**Figure 2:**
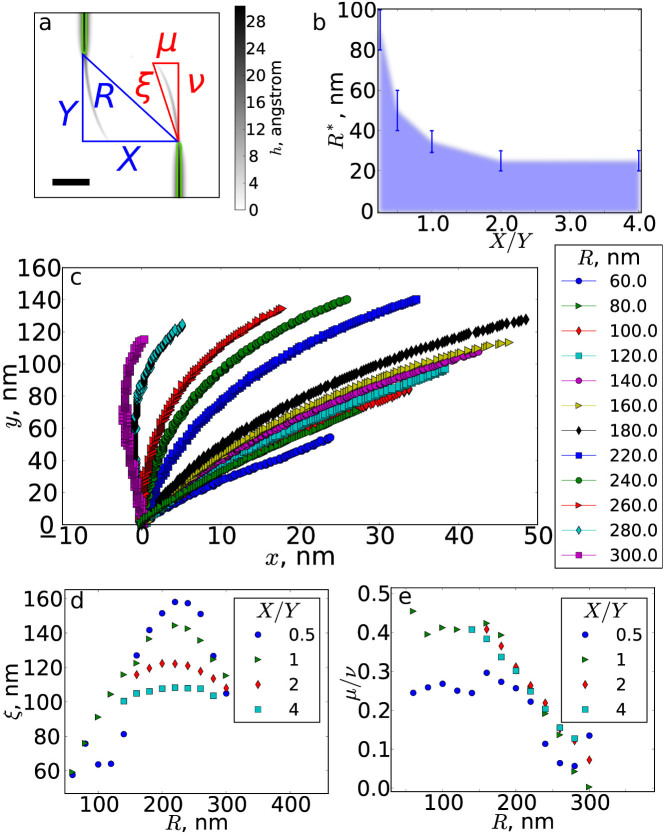
Avoidance and morphology of growing wrinkles have universal dependence on initial separation. (a) Schematic of controlled wrinkle interaction tests, shown on a close-up of the central region of a sheet. Elongated “wrinkle nuclei”, indicated by green lines, are lifted with constant force, to cause wrinkles to propagate towards the centre of the sheet. We investigate how changing the nucleus separations *X* and *Y* affects the wrinkle shape, which is characterized by *μ* and *ν*. The scale bar indicates 50 nm. (b) Whether two wrinkles join depends on nucleus separations, here characterized by aspect ratio *X*/*Y* and distance *R*. When the nuclei are nearly head on (*X*/*Y* small), even distant pairs join, but the maximum distance for joining drops off rapidly as the aspect ratio is increased. (c) Wrinkle shapes for *X* = *Y*, for a range of *R* values. The wrinkle nucleus is excluded. (d) The extension of a wrinkle, 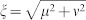, is maximised for a separation of ~220 nm, but decreases with increasing aspect ratio. (e) Remarkably, when *X*/*Y* ≥ 1, the aspect ratio of the wrinkle, *μ*/*ν* depends only on nucleus tip separation *R*.

We first study the growth and interactions of wrinkles, with a focus on avoiding pairs. In order to characterize these pairs, we require a controlled method for creating and growing them. Inspired by studies of interacting cracks grown from a pair of notches^21^, we study propagating and interacting wrinkles using simulations in which an initially flat sheet of dimensions 600 × 600 nm^2^ is subject to upward pulling in “wrinkle nuclei” regions. The tips of the nuclei are separated by distances *X* and *Y*, as defined in Fig. 2(a), which are our control parameters. Because the nuclei are long and narrow and the sheet has high stretching cost, the upward driving primarily causes the wrinkle to propagate forward, with minimal lateral broadening, as shown in Fig. 2(a). We run each simulation until a stable configuration is attained, which occurs when the applied upward forces are balanced by stresses in the sheet.

Whereas an isolated wrinkle propagates directly forward, parallel to its nucleus, nearby wrinkles interact and either join or form an avoiding pair. To uncover the role of interactions, we fix driving conditions and make a systematic study of the effect of wrinkle nucleus separation, which we characterize by aspect ratio *X*/*Y* and distance 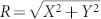. As shown in Fig. 2(b), when *X*/*Y* > 1, the joining condition is simply a distance threshold of ~30 nm. However, for *X*/*Y* < 1, wrinkle merging becomes more favourable and the minimum distance required for the formation of an avoiding pair increases rapidly.

### Wrinkle morphology is controlled by stress fields

In addition to the conditions for avoiding pair formation, we also characterize how the morphology of such pairs depends on wrinkle nuclei separation. Typical wrinkle shapes are shown in Fig. 2(c), for fixed *X*/*Y* = 1. For small *R*, wrinkles are directed toward each other, but for larger *R* they are initially repelled before being attracted. To quantitatively describe the wrinkle shapes, we focus on two characteristics which are indicated in Fig. 2(a): wrinkle extension, which is the distance 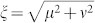 from base to tip, and aspect ratio *μ*/*ν*. For fixed *X*/*Y*, *ξ* depends on *R* and is maximized at a distance ~230 nm, as shown in Fig. 2(d). As shown in Fig. 2(e), the maximum extension decreases with increasing *X*/*Y*, and avoiding pairs of wrinkles formed by nearly head-on nuclei are substantially longer than wrinkles formed from nuclei that are separated in the *X* direction. Remarkably, for nucleus separations 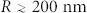, *X*/*Y* has little effect on the aspect ratio *μ*/*ν* of wrinkles, which decreases approximately linearly with *R*.

**Figure 3:**
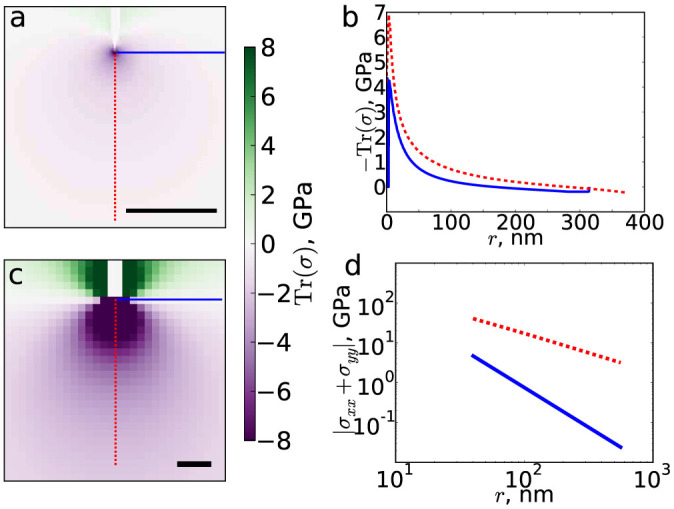
Wrinkle tips generate anisotropic stress fields. (a) Trace of the stress tensor from a single wrinkle, showing tension behind the tip and compression in front, concentrated at the tip. The scale bar represents 100 nm. (b) Slices of the stress trace parallel and perpendicular to the wrinkle direction, as indicated by red and blue lines in panel (a). (c) *σ_xx_* + *σ_yy_* for an infinite sheet with an idealized wrinkle imposed. (d) Slices of *σ_xx_* + *σ_yy_* parallel and perpendicular to the wrinkle direction, as indicated by red and blue lines in panel (c). For clarity, in panels (a) and (c) the interiors of the wrinkles, defined by a height threshold of 2 Å, are not shown.

To understand how wrinkle interactions affect their shape, we characterize the stresses induced by wrinkles, using the trace of the stress tensor Tr(*σ*) = *σ_xx_* + *σ_yy_* + *σ_zz_*. Unlike, e.g, tensile fracture, where hoop stress *σ_θθ_* determines crack propagation^29^, the symmetry of wrinkle propagation makes the isotropic stress the relevant quantity. We first consider a single wrinkle. As shown in Fig. 3, stresses emanating from the tip of a wrinkle are compressive (Tr(*σ*) < 0). The wrinkle grows *via* buckling of the sheet, and deformation occurs primarily as bending rather than stretching which is highly unfavourable for graphene.

As indicated in Fig. 3(b), the stress distribution displays an angular dependence and compressive stresses are largest along a line parallel to the wrinkle. The stress decays rapidly, faster than a power law. We have also calculated the stresses generated by an idealized wrinkle in an infinite sheet under the assumption of zero in-plane deformation by imposing a wrinkle height profile 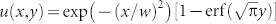 and numerically calculating the stresses using the Föppl-von Kármán equations^30^, with Youngs modulus *E* = 2.4 TPa (Ref. 31, 32). As shown in Fig. 3(c,d), the idealized wrinkle also generates a stress field with an angular dependence. Far from the wrinkle tip, the stresses decay as a power law with exponent 1 parallel and 2 perpendicular to the wrinkle axis.

**Figure 4:**
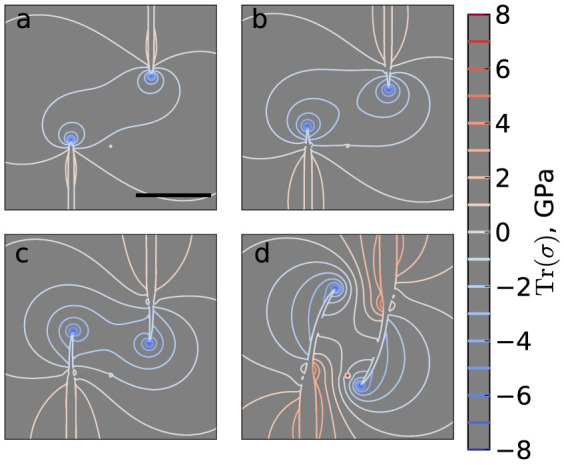
Wrinkle propagation is controlled by isotropic stresses. (a–d) Contour lines of the trace of the stress tensor outside the wrinkles during the evolution of an avoiding pair of wrinkles, with a field of view that excludes the sheet edges. The nucleus separations are *X* = *Y* = 56.6 nm. The line joining the wrinkle tips is a local maximum in the stress distribution and the tips propagate around the centre of this line, which is a saddle point. For clarity, the interiors of the wrinkles (defined by a height threshold of 2 Å) are not shown. All images have the same scale; the scale bar in panel (a) represents 100 nm.

When two wrinkles are grown in proximity, their behaviour is determined by the superposition of their stress fields. A video showing the evolution of stresses for a growing pair of wrinkles is provided as Supplementary Information. As shown in Fig. 4, wrinkles propagate along the steepest stress gradient in front of them. Pairs of wrinkles propagate in a spiral around the centre of the line joining their tips. As seen in Fig. 4(a,b), wrinkle tips initially propagate outwards slightly, and the high-stress central region expands. As the tips pass each other, their motion changes to an inwards propagation, as seen in Fig. 4(c), and the high-stress region contracts. The wrinkles continue to propagate around the centre of the line joining their tips, and come to a stop in the configuration seen in Fig. 4(d). This configuration is stabilized by the balance between applied forces and stresses in the sheet. The deformations required to merge two wrinkles are highly unfavourable and avoiding pairs are hard to remove. As seen in Fig. 2(d), when wrinkle nuclei are close, wrinkles are short because they are unable to grow far before blocking each other. On the other hand, for large nuclei distances, stresses due to interactions are reduced and wrinkle propagation is decreased accordingly. The existence of an optimal tip distance for wrinkle growth is due to the competition between these factors.

### Friction suppresses wrinkle formation

We also test how wrinkle propagation is affected by friction. We take two complementary approaches to modelling frictional effects, which give remarkably similar results, despite one approach being much simpler than the other. In agreement with intuition, we find that friction hampers the propagation of wrinkles.

**Figure 5:**
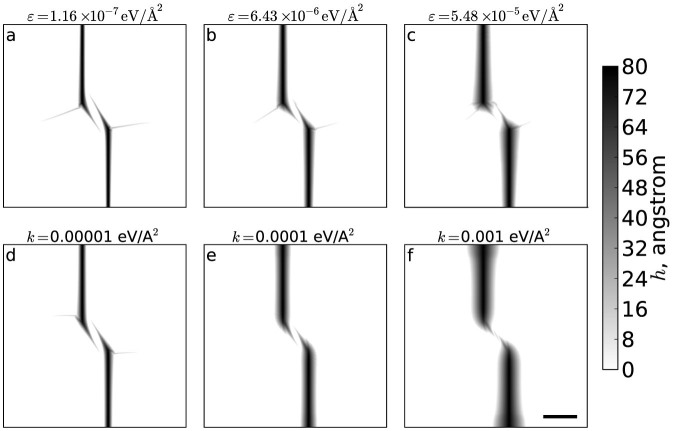
Friction suppresses wrinkle propagation by preventing sliding. Pairs of wrinkles subject to friction implemented either as (a–c) a substrate with random pinning or (d–f) effective friction through a harmonic coupling of the edges of the sheet with their initial lateral positions. *ε* values refer to the measured strain energy density in the sheet once it has relaxed on the randomized substrate, *k* values refer to the spring constant of the harmonic coupling of the sheet edges. In all cases the wrinkle nuclei are separated by *X* = *Y* = 84.8 nm. The scale bar in (f) indicates 100 nm.

The first approach is to introduce quenched disorder on the substrate to induce pinning and friction. Substrate characteristics are given in Table S1. Figure 5(a–c) shows how this disorder affects propagation of wrinkles. In the limit of low friction, wrinkles propagate a long distance without the wrinkle nuclei growing substantially. As friction is increased, wrinkle growth is reduced and the response to the upward pulling of the nuclei is dominated by detachment of the nuclei.

We have also tested the effective friction used in our simulations presented in this paper, by varying the strength of harmonic coupling of the edges of the sheet with their original lateral positions, for a sheet in a Lennard-Jones potential without random pinning. The stronger the coupling, the larger the effective friction, as the cost of sliding of the sheet increases. In the limit of very stiff coupling, for the sheet to buckle out of plane it must stretch, a mechanism which is otherwise avoided due to large energetic costs. As seen in Fig. 5(d–f), increasing the coupling is qualitatively the same as increasing substrate pinning. The coupling *k* = 0.0001 eV/Å^2^, shown in Fig. 5(e), corresponds to the value used in the other simulations reported in this paper.

### Wrinkles across length scales: simulations of multilayers

An advantage of coarse grained simulations is that sheet thickness enters as a change in energetics, without a change in the number of particles in the system, so that multilayer sheets can readily be simulated. Bending costs grow faster than stretching costs with sheet thickness: bending energy scales as *n*^3^ and stretching as *n*, where *n* is the number of layers. Therefore, the minimum feature size of a sheet's morphology increases with its thickness. We examine the consequences of this through simulations of multilayer graphene sheets, which serve as a bridge between micro and macro length scales. We consider two scenarios: interacting growing wrinkles and deposition on patterned substrates.

**Figure 6:**
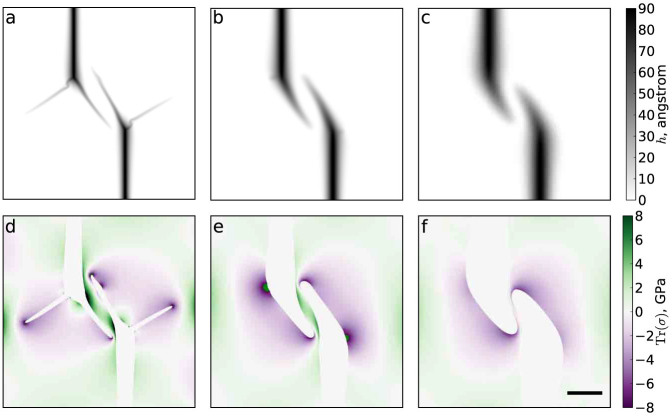
Wrinkle interaction tests on multilayers illustrate how avoiding pairs evolve with sheet thickness. Shown here are height maps for (a) 2-, (b) 5-, and (c) 10-layer graphene sheets. Panels (d–f) show the corresponding trace of the stress tensor outside the wrinkles. To make the shapes of wrinkles clearer, their interiors (defined by a height threshold of 2 Å) are left white. The nucleus separations are *X* = *Y* = 160 nm. All images have the same scale; the scale bar in panel (c) represents 100 nm.

Figure 6 illustrates how wrinkle configurations change as sheet thickness is increased, up to 10 layers. We remark on how the wrinkles in the 10-layer sheet are reminiscent of those seen in geomembranes (see Fig. 1(i,j)). In general, wrinkles become shorter and wider with increasing sheet thickness, and in thick sheets wrinkle nuclei broaden considerably during wrinkle growth. Stresses in the sheet are reduced as its thickness increases, as shown in Fig. 6(d–f). Similar results are also seen in simulations of deposition of mono- and multilayers on substrates decorated with four particles (Fig. S5).

## Discussion

We have presented a systematic study of the interactions and coalescence of delamination patterns in thin sheets, as exemplified by wrinkles in graphene on patterned substrates. We have shown how the morphology of pairs of wrinkles is determined by their interactions, which are mediated by stresses in the sheet.

Delamination is a form of fracture, so it is not surprising our interacting delamination wrinkles are reminiscent of interacting cracks. However, a fundamental geometrical difference exists between the wrinkle and crack scenarios. Wrinkles can move laterally without broadening, whereas in the absence of healing cracks can only grow at their tips or by opening up. In this sense, crack propagation is “irreversible”, whereas wrinkles can move around to reduce stresses, as seen in example wrinkle propagation movies in the Supplementary Information. This introduces a subtlety into the analysis of wrinkle shapes, because their final shape is not necessarily a trace of the motion of their tips.

Experiments on interacting cracks in gelatine plates^21^ reveal a universal shape for the lenticular region cut out by two interacting cracks, described by 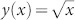. In those experiments, notch lateral separation (equivalent to our *X*, see Fig. 2(a)) was varied over an order of magnitude, with fixed separation along the direction of the notches (equivalent to our *Y* in Fig. 2(a)). By way of contrast, we are unable to identify a universal shape in our data on avoiding wrinkle pairs, as is obvious from visual inspection of Fig. 2(c), in which some wrinkles are almost straight and others are substantially curved. Furthermore, in those experiments, cracks were found to only deviate from straight paths when their tips pass each other, whereas we find wrinkles that are curved even at their nuclei. We attribute these differences in behaviour to differences in the stress fields, which in our simulations are affected by system edges, as seen for example in Fig. 3. We also note that wrinkle morphologies may be affected by the initial orientations imposed by the wrinkle nuclei geometry.

Although edge effects in our simulations make it difficult to directly apply our quantitative results to larger systems, open boundary conditions are essential for qualitatively correct results. As shown in Fig. S3, periodic boundary conditions fix the overall dimensions of the sheet and prevent sliding, so that out of plane buckling requires costly stretching and wrinkles are not observed. The importance of open boundary conditions raises questions about a recent simulation study of delamination wrinkles connecting pairs of nanoparticles^18^, since that study used periodic boundary conditions along the direction of the nanoparticle centre-centre line, which may have a substantial and uncontrolled effect on wrinkle morphology.

Control of cracks by interactions is also seen in experiments on cracks in paper that are grown by pulling paper in a direction perpendicular to two lines of slits^33^. The slits and their spacing are fixed, and the control parameter is the distance *d* between the two lines. Because of disorder and complex interactions between the several cracks present in the system, crack growth is probabilistic. For small *d*, cracks typically repel each other during their initial growth, before becoming attracted. For larger *d*, repulsion is seen less frequently, until a transition inter-line distance above which cracks from different lines do not interact. This is consistent with the behaviour seen in wrinkle shape data for *X*/*Y* = 1 in Fig. 2(c).

We have shown that wrinkle interactions and growth are hampered by friction between sheet and substrate. Such friction would be relevant for experiments on sheets bonded to substrates. For example, in Ref. 34, the strain energy of a graphene sheet adhered to a substrate was found to be ~10^−3^ eV/Å^2^. This is two orders of magnitude larger than the strain energy due to pinning on the highest-friction substrate we have simulated (see Fig. 5). However, in a deposition scenario such as that studied by Yamamoto *et al.*^17^, frictional effects are likely lower because the sheet deforms before it is fully in contact with the substrate. This low-friction scenario is consistent with the long smooth wrinkles they observe (see Fig. 1 and Ref. 17). Movies of our deposition simulations indicate that wrinkles form as soon as the sheet makes contact with the particle tops, well before it touches the lower substrate. By way of contrast, atomistic simulations of deposition with strong friction, reported in Fig. S4, do not display such wrinkles.

Our results are important for controlling the morphology of graphene sheets for applications, but are not specific to graphene and are applicable for other thin films. Avoiding pairs of wrinkles are a feature found even on the length scales of geomembranes. Accordingly, we expect our results to be experimentally testable not only in graphene but in tabletop experiments on other thin sheets.

## Methods

### Coarsegrained model

The graphene sheet is modeled as a triangular lattice of particles connected by linear springs with rest length *a* = 5 Å and stiffness 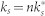, where *n* is the number of graphene layers in the sheet and 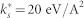
31,32. Bending rigidity is implemented as an energy that depends on the angle *φ* between neighbouring triangles and is minimized when the sheet is flat: *E*_bend_ = *k_b_*(*φ* − *π*)^2^ where 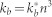 is the bending stiffness with 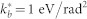
35,36. The simulation uses the molecular dynamics package LAMMPS^37^ and is conducted at zero temperature, with damping applied to slowly drain energy from the system. Further details are given as Supplementary Information.

The sheet is pushed downward with a constant position-independent force of 0.001 eV/Å to simulate deposition. The substrate acts as a barrier to the sheet. For computational efficiency, in most simulations we model the interaction with the substrate as harmonic repulsion, that is, the energy of a particle with radial distance *r* from the substrate is
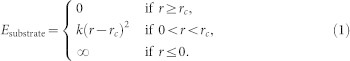
where *k* = 1.0 eV/Å^2^ is a spring constant and *r_c_* = 1 Å. We have also tested a radial Lennard-Jones potential with parameters to match experiments (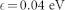, *σ* = 0.1 nm^17^) and find the details of substrate repulsion do not affect wrinkle morphology, as shown in Fig. S2.

Open boundary conditions are used, so the sheet can expand and shrink laterally. Particles within a few nm of the sheet edge are coupled to their original lateral positions with harmonic springs, of spring constant *k* = 0.0001 eV/Å^2^, unless otherwise stated. As shown in Fig. 5, this method compares favourably with a more computationally intensive model of a substrate with random pinning and effectively prevents the sheet sliding, which is the most important effect of friction here.

### Wrinkle interaction tests

We place a sheet on a flat substrate, with substrate interaction given by Eq. (1) and the same parameters as above. A downward force of 0.001 eV/Å is applied on the whole sheet. We apply an upward force of 0.01 eV/Å inside the wrinkle nuclei, except for the multilayer simulations, in which the upward force is 0.1 eV/Å. The nuclei are rectangular regions of width 32 Å with rounded tips, that extend to the sheet edge. We have also tested simulations where the upward force is increased slowly, rather than turned on instantaneously, and find this makes little difference to final configurations. We have also tested changing the nucleus orientation relative to the sheet edges (and therefore lattice), but do not find any lattice effects.

### Substrates with random pinning

Our simulations of substrates with random pinning use a Lennard-Jones potential for the substrate, with parameters to match experiments (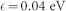, *σ* = 0.1 nm^17^). We do not apply an external downward force in this case. Random pinning is implemented as randomly-positioned Gaussian potentials of random depth, both positive and negative. The Gaussian potentials have the form *A* exp(−*Br*^2^) for *r* < 1.01 Å, with *A* drawn from a uniform random distribution over [−5, 5) eV and *B* = 10 Å^−2^. The centres of these lie on a plane at distance *z*_0_ below the minimum of a Lennard-Jones potential. The LJ potential is the dominant contribution so that in the absence of external forces the graphene sheet sits near the minimum of the LJ potential. Varying *z*_0_ tunes the strength of the random contributions to the sheet's energy.

## Author Contributions

S.Z. designed the research. Zoe B. and A.L.S. performed simulations. Zoe B., A.L.S., Zsolt B. and S.Z. analyzed data. Zoe B. wrote the paper with input from all authors.

## Supplementary Material

Supplementary Information four_particles_spacing160nm_1layer

Supplementary Information four_particles_spacing160nm_5layers

Supplementary Information wrinkle_pair_const-force

Supplementary Information wrinkle_pair_ramp-up

Supplementary Information wrinkle_pair_StressXYZ_ramp-up

Supplementary Information Supplementary Information for 'Wrinkle motifs in thin films'

## Figures and Tables

**Table 1:** Avoiding wrinkle pairs exist on nanometre to metre length scales. Examples of avoiding pairs of wrinkles in the literature, sorted by sheet thickness. This is not intended to be an exhaustive list, but rather to give a sense of the variety of situations in which avoiding pairs occur

Reference	Figure	Material	Substrate	Thickness	Wrinkle width
[17]	Fig. 1d,e. a	Graphene	Silicon/silica nanoparticles	Single atom	~7.4 nm b
[39]	Fig. 2b, upper centre.	Graphene (exposed to O_2_)	Ru(1000)	Single atom	~1 nm
[2]	Fig. 2b, lower right panel.	In_0.2_Ga_0.8_As	AlAs with pits	10 nm	~1 *μ*m
[40]	Fig. 2b, e.g, near upper edge of scale bar.	C	Glass	80 nm	~5 *μ*m
[6]	Fig. 1b.	ZnO	Silicon	85 nm	~0.5 *μ*m
[5]	Fig. 1a.	Ni	Polycarbonate	350 nm	~10 *μ*m
[41]	Fig. 5, near image centre.	High-density polyethylene	Clay	1.5 mm	0.31 ± 0.06 m c

^a^c.f. Fig. 1 of main text.

^b^Based on the average wrinkle width being approximately the nanoparticle diameter^17^.

^c^As quoted in Ref. 41.
