# Supplementary material for: Wrinkle motifs in thin films
Source: Sci Rep. 2015 Mar 11;5:8938. doi: 10.1038/srep08938 (PMC4355740; doi:10.1038/srep08938)
Supplement: Supplementary Information — for 'Wrinkle motifs in thin films' [file srep08938-s6.pdf]

Supplementary Information for ‘Wrinkle motifs in thin films’

Zoe Budrikis,[1] Alessandro L. Sellerio,[2] Zsolt Bertalan,[1] and Stefano Zapperi[2,1]

[1] *ISI Foundation, Via Alassio 11/c, 10126 Torino, Italy*

[2] *CNR-IENI, Via R. Cozzi 53, 20125 Milano, Italy*

Supplementary text

Implementation details for coarse grained model

We implement our simulations using the molecular dynamics package LAMMPS [1]. Particles are initially generated in a flat triangular lattice. They are connected by harmonic bonds using the `bond_style harmonic` command, to give a stretching cost to the sheet, as described in the main text. The bending cost of the sheet is implemented using improper interactions between sets of four particles on the vertices of two neighboring triangles, using the `improper_style harmonic` command.

The substrate consists of a flat repulsive sheet, which is implemented as a `plane` which interacts with the particles through a `wall/region` fix, with a harmonic interaction used. Only the repulsive part of the harmonic potential is used, and we have found similar results using a Lennard-Jones interaction. The nanoparticles are implemented using the `indent` fix (spring constant 1.0 eV/Å^2), which allows the nanoparticles to deform slightly under the influence of the sheet. This avoids errors in which the particles of the sheet intersect with the nanoparticle region, which we found to be a problem when using a `wall/region` fix for the nanoparticles.

We implement an effective friction with the substrate by defining a `group` of edge atoms at the simulation start, and using a `spring/self` fix to couple the atoms to their initial lateral positions using springs of stiffness of stiffness 0.0001 eV/Å^2 (no vertical forces are applied). The edge region typically consists of the atoms outside a square with side length $\sim$ 10 nm less than the sheet side length, but we do not see any sensitivity to the details of this except in very small sheets.

In simulations of substrates with random pinning, we implement the substrate as a Lennard-Jones potential (with $\epsilon = 0.04$ eV, $\sigma = 1$ Å and a cutoff at 2.5 Å), with Gaussian potential wells and bumps placed at random positions on a plane below the minimum of the LJ potential. By varying the position of this plane, we are able to tune the strength of the random potential, and if the plane is not brought too close to the LJ minimum the mean adhesion force experienced by the sheet is not affected. The Gaussian potentials have the form $A\exp(-Br^2)$ for $r < 1.01$ Å, with A drawn from a uniform random distribution over $[-5, 5)$ eV and $B = 10$ Å^{-2}, and are placed uniformly at random with a density of 0.1.

The strength of friction is quantified by the strain energy per area of the sheet once it has relaxed in this potential, without any external forces applied.

We drain energy from the system with a `viscous` fix with damping parameter 5×10^{-4} eV/ps. We run our simulations until no change in sheet configuration can be seen in two snapshots separated by 10^4 time steps. We use a time step of 0.0005 ps.

The stress on particle i is given by the tensor

$$S_{\alpha\beta}^{(i)} = -\Bigg[m v_\alpha^{(i)} v_\beta^{(i)} + \frac{1}{2}\Big(N_B^{(i)} r_\alpha^{(i)} F_\beta^{(i)} + \sum_{n=1}^{N_B^{(i)}} r_\alpha^{(n)} F_\beta^{(n)}\Big) + \frac{1}{4}\Big(N_I^{(i)} r_\alpha^{(i)} F_\beta^{(i)} + \sum_{n=1}^{N_I^{(i)}} \sum_{j=1}^{3} r_\alpha^{(j)} F_\beta^{(j)}\Big)\Bigg]. \tag{S1}$$

The first term is a contribution from the kinetic energy, which is zero at the end of our simulations, when we measure the stress. The second term gives contributions from each of the $N_B^{(i)}$ bonds particle i is a member of, and the final term gives contributions from the $N_I^{(i)}$ impropers particle i is a member of. $r^{(i)}$ is the position of particle i and $F^{(i)}$ refers to the force on i as a result of the relevant interaction. The quantity S given by Eq. (S1) is not strictly a stress, but is multiplied by a characteristic volume and is returned with units bars Å^2. We obtain the stress σ on the sheet by taking the particle volume to be the area of a triangle (here ~ 10.8 Å^2), multiplied by the sheet thickness, which is approximately $3n$ Å. We focus on the stress trace in part because it is invariant and therefore independent of the local orientation of the sheet.

Nature of substrate radial interactions

As discussed in the main text, in our simulations the substrate exerts a harmonic repulsion on the graphene sheet, with a potential energy of the form

$$E_{\text{harmonic}} = \begin{cases} 0 & \text{if } r \geq r_c, \\ k(r - r_c)^2 & \text{if } 0 < r < r_c, \\ \infty & \text{if } r \leq 0. \end{cases} \tag{S2}$$

where r is the radial distance from the substrate, $k = 1.0$ eV/Å^2 is a spring constant and $r_c = 1$ Å. In addition to this interaction, in order to push the sheet towards the substrate

a constant downward force of $f_{\rm down} = 0.001$ eV/Å is applied to each particle in the sheet throughout the simulation. Figure S1 shows the potential energy as a function of height above the substrate for a flat sheet. Minimization of the total potential energy gives an equilibrium height above the substrate of $r_c - f_{\rm down}/(2k) = 0.9995$ Å.

We have also tested a Lennard-Jones substrate interaction. In that case, the interaction with the substrate has potential energy

$$E_{\rm LJ} = 4\epsilon\big[\big(\frac{\sigma}{r}\big)^{12} - \big(\frac{\sigma}{r}\big)^{6}\big], \tag{S3}$$

with $\epsilon = 0.04$ eV and $\sigma = 1$Å. In those simulations, a position-independent downward force of 0.001 eV/Å is applied for the first 5×10^5 simulation steps (out of typically 10^6 required for full equilibration), and is then turned off. The potential energy as a function of height above a substrate for these simulations is shown in Fig. S1.

Although the energy minimum near a height of 1Å is more pronounced for the LJ potential, as seen in Fig. S1, we find this makes little difference to simulations. Figure S2 shows height maps for simulations of a monolayer sheet deposited on a substrate with four particles arranged in a square with side length 140 nm, for the two types of substrate interactions. The differences between the two configurations are minimal, and importantly the wrinkle wavelengths are unaffected.

Friction between sheet and substrate

As discussed in the main text, we have studied a substrate with random pinning to model friction. This is implemented as randomly-positioned Gaussian potentials whose centers are on a plane at a distance z_0 below the minimum of a Lennard-Jones potential. The LJ potential is the dominant contribution so that in the absence of external forces the graphene sheet sits near the minimum of the LJ potential. Varying z_0 enables us to tune the strength of the random contributions to the sheet's energy.

We make two characterizations of the potential. (i) The mean strain energy of a sheet that has relaxed in the potential, ε. This is exponentially distributed, so we also give the maximum values measured, $\varepsilon_{\rm max}$ (the minimum values are always ~ 0). (ii) The mean adhesion energy of the relaxed sheet, Γ. Table S1 reports these characteristics for the three substrates we study in the main text.

Atomistic simulations: Boundary conditions and importance of sliding

To see the effects of boundary conditions and friction for graphene wrinkling, performed molecular dynamics simulations of a single layer, monocrystalline graphene patch composed of approximately 570,000 atoms with dimensions 140×140 nm^2, interacting with a substrate. The geometry of the substrate consists of a flat plane with two spherical particles on top and the interaction between graphene and substrate was simulated in two different ways. In the first, the substrate is a continuous wall that interacts with the carbon atoms *via* a radial Lennard-Jones potential with parameters $\epsilon = 0.04$ eV, $\sigma = 0.1$ nm, and a wall-atom long-range cutoff distance ($d = 2.5$ nm). These were chosen to match the C–SiO_2 interaction, as indicated in [2]. In this case, there is no friction between the sheet and substrate because the forces are purely perpendicular to the surface. The second substrate consists of a Si monocrystal with approximately 3 million atoms. The pairwise atomic interactions are hybrid. C–C interaction uses the AIREBO potential [3], which has been extensively used to simulate and predict mechanical properties of carbon-based materials, i.e. fullerenes, carbon nanotubes and graphene [4]. C–Si interactions are defined by the “Tersoff” pairwise potential, as described in Ref. [5]. In this case, the atoms of the substrate are not time-integrated (they remain frozen). In this case the friction between sheet and substrate is large, due to the strong coupling between C and Si atoms.

The graphene layer is thermalized using a Berendsen [6] thermostat set at 300K, with a characteristic relaxation time set to 0.1 ps; the simulation timestep is set at 1 fs to ensure a correct time integration of the atom dynamics. All simulations were performed using the LAMMPS molecular dynamics toolbox [1]. The simulation protocol consists of four steps. (i) the graphene layer is generated, and the system is brought to the energy minimum using conjugate gradient minimization; (ii) the graphene layer is placed above the substrate, just beyond the cutoff of the potential, and the atoms are given a small initial velocity towards the substrate; (iii) the graphene interacts with the substrate and becomes attached to it; the simulation is run until the whole patch has come in contact; in some cases (PBC), periodic oscillations around an equilibrium configuration can be observed; the thermostat induces only minimal damping, and the oscillations can be observed for long times; (iv) we drive the system to its equilibrium configuration by removing the thermostat and by adding an artificial viscous damping term. The simulation is stopped once a lower threshold in atom

motion is reached.

As seen in Fig. S3, periodic boundary conditions result in a very smooth deformation of the sheet, which remains flat and attached to the substrate away from the particle pair. We attribute this to the fact that the total dimensions of the sheet are fixed. This places a strong constraint on out of plane buckling which must, in this case, involve costly stretching. On the other hand, open boundary conditions allow wrinkles to form not only as a delaminated region between the particles, but also away from the particles, in a manner reminiscent of that seen in the experiments of Yamamoto *et al* [2].

The necessity of open boundary conditions for realistic results also points to the importance of allowing sliding in the graphene sheet in the scenarios we study here. This can be seen in atomistic simulations of graphene which interacts with a crystalline silicon substrate via Tersoff interaction, which is rather strong. A typical configuration is shown in Fig. . The graphene adheres strongly on contact and once adhered, can neither detach nor move laterally. As a result, although wrinkles are present in the graphene, they are lower than those observed experimentally and are atomically narrow. We believe they correspond to boundaries between regions of graphene that have adhered to the substrate in an incompatible way, and are therefore fundamentally different in origin to the wrinkles observed in experiments [2] and our model.

Delamination of graphene multilayers on particle-decorated substrates

We have studied the role of wrinkle coalescence in the transition from conformation to delamination of a sheet deposited on a substrate decorated with nanoparticles. Previous simulations of deposition on small clusters of nanoparticles indicate interesting pseudomagnetic field distributions [7], which provide technological motivation for these studies. We simulate a 600×600 nm^2 graphene sheet, on a substrate with four nanoparticles of diameter 8 nm, arranged in a rectangle with aspect ratio close to unity and a range of side lengths s, with centre close to the sheet centre. (We use this configuration, rather than a square centred exactly, in order to avoid spurious effects arising from symmetry.) Example movies are provided (`four_particles_spacing160nm_1layer.avi` and `four_particles_spacing160nm_5layers.avi`).

Figure S5(a–d) shows typical configurations for close-spaced and distant particles, for a

monolayer (panels a and b) and a 5-layer sheet (panels c and d). When particles are close together, the sheet detaches in the centre of the rectangle. As particle spacing is increased, the sheet conforms to the substrate except at wrinkles that radiate from the particles. Increasing the sheet thickness 'smooths' the sheet and increases the size of features, as seen by comparing panels (a,b) and (c,d) of Fig. S5. Thicker sheets also have lower stresses, as seen in Fig. S5(e–h). Near the particles, stresses are tensile ($\mathrm{Tr}(\sigma) > 0$), but compressive regions exist inside wrinkles and especially at the wrinkle tips.

The delamination transition is quantified in Fig. S5(i), which shows the height of the centre of the sheet as a function of particle spacing s, for 1-, 2-, 5- and 10-layer sheets. In all cases, a sharp transition is observed at a particle spacing s^* that depends on sheet thickness. A first estimate of this dependence is given by assuming bending rigidity does not enter the problem and the sheet deforms purely through stretching. Then the delaminated region of an n-layer sheet around a protrusion has radius $R \approx r(4nE_{2D}/3\Gamma)^{1/4}$ [2, 8], where r is the particle radius, E_{2D} is the tensile rigidity of the sheet and Γ characterizes the adhesion energy. Under the assumption that the sheet detaches when delaminated regions meet, the critical particle spacing scales as $s^* \sim n^{1/4}$. As seen in Fig. S5(j), this scaling collapses the 1- and 2-layer curves well, but already breaks down for the 5-layer sheet. This is unsurprising, because the argument does not take into account the existence of stress fields and wrinkles connecting the particles.

[1] Plimpton, S. Fast parallel algorithms for short-range molecular dynamics. *J. Comp. Phys.* **117**, 1–19 (1995).

[2] Yamamoto, M., Pierre-Louis, O., Huang, J., Fuhrer, M. S., Einstein, T. L., and Cullen, W. G. "The Princess and the Pea" at the Nanoscale: Wrinkling and Delamination of Graphene on Nanoparticles. *Phys. Rev. X* **2**, 041018 (2012).

[3] Stuart, S. J., Tutein, A. B., and Harrison, J. A. A reactive potential for hydrocarbons with intermolecular interactions. *The Journal of Chemical Physics* **112**(14), 6472–6486 (2000).

[4] Zhao, H., Min, K., and Aluru, N. R. Size and chirality dependent elastic properties of graphene nanoribbons under uniaxial tension. *Nano Letters* **9**(8), 3012–3015 (2009).

[5] Tersoff, J. New empirical approach for the structure and energy of covalent systems. *Phys.*

Rev. B **37**, 6991–7000 (1988).

[6] Berendsen, H. J. C., Postma, J. P. M., van Gunsteren, W. F., DiNola, A., and Haak, J. R. Molecular dynamics with coupling to an external bath. *The Journal of Chemical Physics* **81**(8), 3684–3690 (1984).

[7] Neek-Amal, M., Covaci, L., and Peeters, F. M. Nanoengineered nonuniform strain in graphene using nanopillars. *Phys. Rev. B* **86**, 041405 (2012).

[8] Zong, Z., Chen, C.-L., Dokmeci, M. R., and Wan, K.-t. Direct measurement of graphene adhesion on silicon surface by intercalation of nanoparticles. *Journal of Applied Physics* **107**(2) (2010).

List of supplementary movies

Included are five video files. They are:

1. `four_particles_spacing160nm_1layer.mov` **Wrinkles form during deposition before the sheet is in full contact with the substrate.** The video shows deposition of a monolayer sheet on four particles of diameter 8nm, with interparticle spacing 160 nm.

2. `four_particles_spacing160nm_5layers.mov` **Increasing sheet thickness increases the size of features formed.** The video shows deposition of a 5-layer sheet on four particles of diameter 8nm, with interparticle spacing 160 nm.

3. `wrinkle_pair_const-force.mov` **Evolution of a typical pair of wrinkles subject to constant upward force in their nuclei.** The wrinkle nuclei have tip separation $X = Y = 113.2$ nm.

4. `wrinkle_pair_ramp-up.mov` **Slowly ramping up the upward force on wrinkle nuclei has little effect on the final configuration of the wrinkles.** The wrinkle nuclei have tip separation $X = Y = 113.2$ nm.

5. `wrinkle_pair_StressXYZ_ramp-up.mov` **Wrinkle tips generate compressive stresses, which they propagate along.** The video shows the trace of the stress tensor, $\mathrm{Tr}(\sigma) = \sigma_{xx} + \sigma_{yy} + \sigma_{zz}$, during the evolution of wrinkles growing due to constant upward force. The wrinkle nuclei have tip separation $X = Y = 113.2$ nm.

Supplementary tables and figures

z_0, Å	ε, eV/Å^2	$\varepsilon_{\rm max}$, eV/Å^2	Γ, eV/Å^2
0.4	5.48×10^{-5}	0.0096	0.0037 ± 0.001
0.5	6.43×10^{-6}	0.0026	0.0037 ± 0.00018
0.6	1.16×10^{-7}	0.00013	$0.0037 \pm 3.5 \times 10^{-5}$

TABLE S1: **Characteristics of substrates with random pinning.** By varying the distance z_0 between the plane containing random pins and the minimum of the LJ potential, we can subject the sheet to two orders of magnitude of strain energy ε, while maintaining approximately constant adhesion Γ.

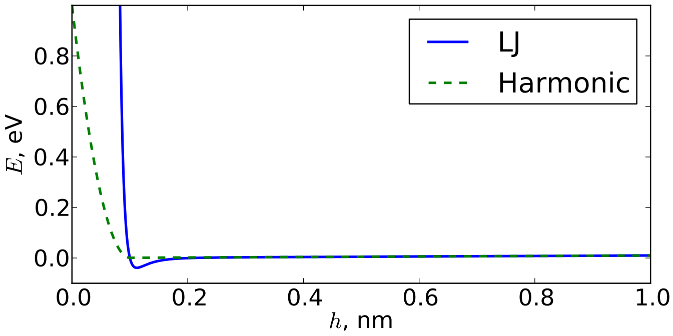

FIG. S1: **A harmonic repulsive substrate with an additional downward force on the graphene sheet acts as a 'cartoon' Lennard-Jones substrate.** Here, we compare the potential energy of a particle at height h above a flat substrate which has either an LJ or a harmonic potential. In both cases, a downward force of $f_{\rm down} = 0.001$ applied in addition to the substrate interactions.

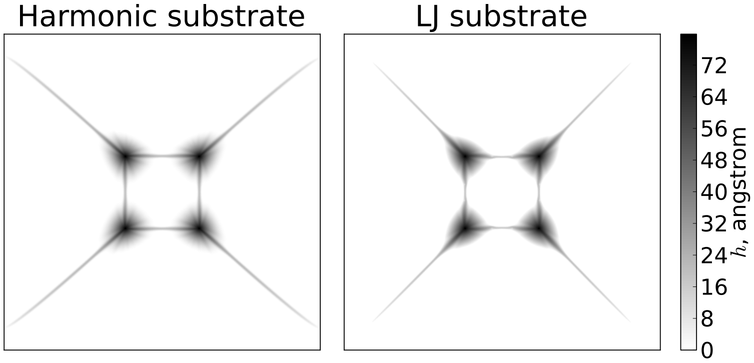

FIG. S2: **Harmonic and Lennard Jones substrates yield similar results in deposition simulations.** In these simulations, a monolayer sheet is deposited on four nanoparticles with particle spacing 140 nm. Left panel: substrate interaction is that used in the simulations in the main text. Right panel: substrate interactions are governed by a Lennard-Jones potential.

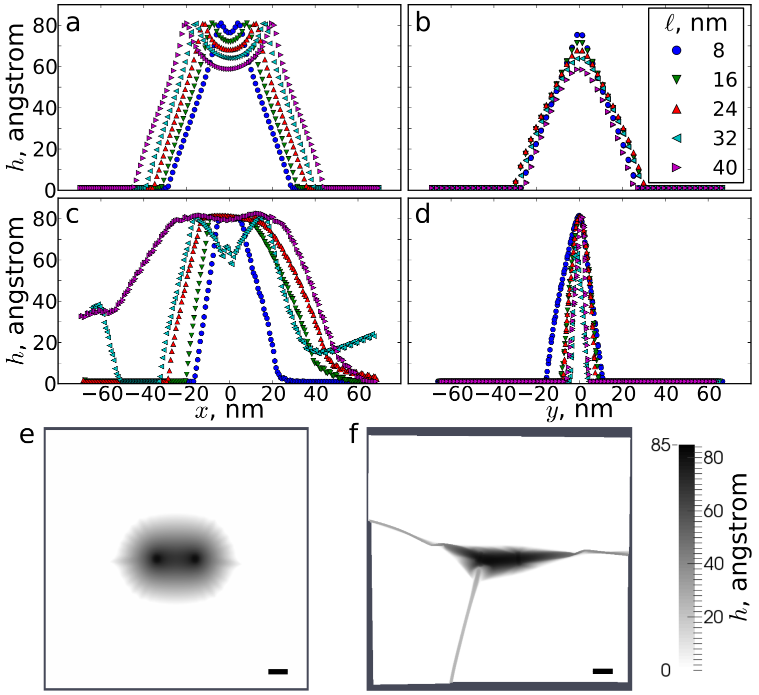

FIG. S3: **Periodic boundary conditions prevent sliding of the sheet and suppress wrinkle formation.** Wrinkle profiles in atomistic simulations, (a) along the x direction and (b) along the y direction, with periodic boundary conditions. (c,d) Corresponding profiles for simulations with open boundary conditions. The graphene sheet is modeled using an AIREBO potential and deposited on a substrate with which it interacts *via* a radial Lennard-Jones potential. The substrate has spherical protrusions of diameter 8 nm which are placed on the x axis (which is parallel to the armchair direction of the graphene) with separation ℓ as indicated in the legend. The lower panels show height maps for configurations obtained with $\ell = 20$ nm, for (e) periodic and (f) open boundary conditions. In panel (f), the contraction of the sheet at the edges can be seen, with the gray regions at the edges representing the substrate. Scale bars represent 10 nm.

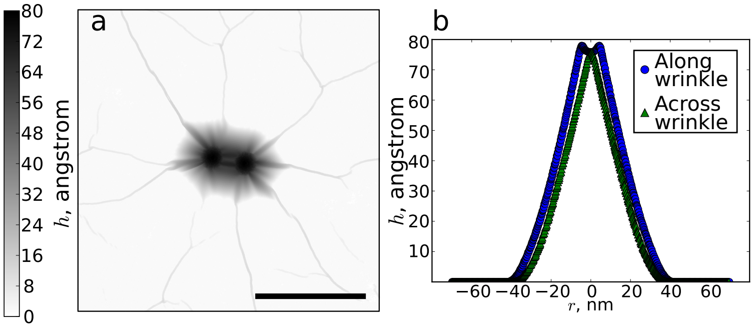

FIG. S4: **Very strong substrate friction prevents wrinkle formation.** Height maps of a monolayer graphene sheet simulated atomistically, with an atomistic Si substrate. The substrate has spherical protrusions of diameter 8 nm and the center-center line of the spheres is at 10° to the x axis (the graphene armchair direction) to ensure the configurations are not affected by spurious lattice effects. The apparent wrinkles are both low and narrow (atomic-scale) and are qualitatively different to the delamination wrinkles we are interested in. The scale bar indicates 50 nm. (b) Corresponding height profiles along lines parallel and perpendicular to the protrusion center-center line.

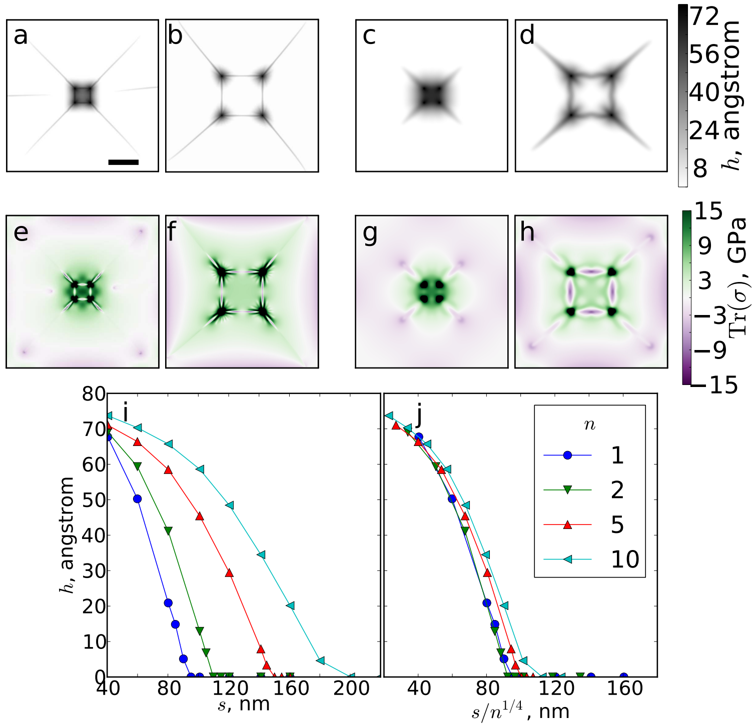

FIG. S5: **Interplay of sheet elasticity and substrate patterning drives a universal delamination transition.** Typical configurations of a sheet deposited on four nanoparticles: (a) monolayer sheet, particle spacing 60 nm, (b) monolayer sheet, particle spacing 160 nm, (c) 5-layer sheet, particle spacing 60 nm, (d) 5-layer sheet, particle spacing 160 nm. Panels (e–h) show the corresponding trace of the stress tensor. Where the stress is above 15 GPa, the color scale has been clipped (black regions on top of the particles). All eight panels have the same scale; the scale bar in panel (a) represents 100 nm. (i) Height of the center of the graphene sheet as a function of particle spacing, for 1, 2, 5 and 10 layer sheets. (j) As a first approximation, the delamination transition occurs at a particle spacing $s^* \sim n^{1/4}$.
